# Individual-level space-time analyses of emergency department data using generalized additive modeling

**DOI:** 10.1186/1471-2458-12-687

**Published:** 2012-08-22

**Authors:** Verónica M Vieira, Janice M Weinberg, Thomas F Webster

**Affiliations:** 1 Program in Public Health, University of California, Irvine, CA, 92697, USA; 2 Department of Environmental Health, Boston University School of Public Health, Talbot 4W, 715 Albany Street, Boston, MA, 02118, USA; 3 Department of Biostatistics, Boston University School of Public Health, Crosstown, 801 Massachusetts Avenue, Boston, MA, 02118, USA

**Keywords:** Geographic information systems, Administrative data, Hospitals

## Abstract

**Background:**

Although daily emergency department (ED) data is a source of information that often includes residence, its potential for space-time analyses at the individual level has not been fully explored. We propose that ED data collected for surveillance purposes can also be used to inform spatial and temporal patterns of disease using generalized additive models (GAMs). This paper describes the methods for adapting GAMs so they can be applied to ED data.

**Methods:**

GAMs are an effective approach for modeling spatial and temporal distributions of point-wise data, producing smoothed surfaces of continuous risk while adjusting for confounders. In addition to disease mapping, the method allows for global and pointwise hypothesis testing and selection of statistically optimum degree of smoothing using standard statistical software. We applied a two-dimensional GAM for location to ED data of overlapping calendar time using a locally-weighted regression smoother. To illustrate our methods, we investigated the association between participants’ address and the risk of gastrointestinal illness in Cape Cod, Massachusetts over time.

**Results:**

The GAM space-time analyses simultaneously smooth in units of distance and time by using the optimum degree of smoothing to create data frames of overlapping time periods and then spatially analyzing each data frame. When resulting maps are viewed in series, each data frame contributes a movie frame, allowing us to visualize changes in magnitude, geographic size, and location of elevated risk smoothed over space and time. In our example data, we observed an underlying geographic pattern of gastrointestinal illness with risks consistently higher in the eastern part of our study area over time and intermittent variations of increased risk during brief periods.

**Conclusions:**

Spatial-temporal analysis of emergency department data with GAMs can be used to map underlying disease risk at the individual-level and view changes in geographic patterns of disease over time while accounting for multiple confounders. Despite the advantages of GAMs, analyses should be considered exploratory in nature. It is possible that even with a conservative cutoff for statistical significance, results of hypothesis testing may be due to chance. This paper illustrates that GAMs can be adapted to measure geographic trends in public health over time using ED data.

## Background

In this paper, we describe methods for applying generalized additive models (GAMs) to retrospectively analyze spatial-temporal illness patterns using existing emergency department (ED) data. GAMs allow for smoothing of data while adjusting for known covariates, thus providing a useful framework for individual-level analyses [1-4]. By dividing the dataset into data frames of overlapping time periods and spatially analyzing each data frame using GAMs, we essentially smooth over time and space. The resulting series of maps, displayed chronologically, create a movie which allows us to visualize changes in magnitude, geographic size, and location of risk.

ED data is routinely collected to identify outbreaks of illness, which necessitates the collection of detailed geographic information, often at the individual address level and reported on a daily basis [5-7]. Even when outbreaks are not occurring, ED data can also be useful for understanding the underlying spatial and temporal distribution of illnesses in the community. GAMs can be used to measure trends for larger geographic areas over longer time periods than is usually associated with acute illness outbreaks. For example, the methods presented here can be used to detect areas of increased illness resulting from persistent contamination of groundwater supplies for municipal drinking water. Our approach using GAMs implemented with standard software offers public health researchers and practitioners an opportunity for exploratory secondary analyses of existing ED data, but other methods may be more appropriate for real-time surveillance of patterns at a smaller scale [7-14].

In addition to the ease of use, the GAM provides some methodological advantages. Selection of separate smoothing parameters for space and time circumvents a common limitation for many space-time methods. Often, clustering is detected in a 3-dimensional analysis where the effect of one unit in 2-dimensional space is forced to be equivalent to the effect of one unit in one-dimensional time despite the units being different [15]. For example, when estimating the risk of disease at a point in space and time, data points one mile away could contribute the same weight to the prediction as points one day apart, which may or may not be appropriate. Our separate treatment of spatial and temporal smoothing is useful and appropriate for exploratory analyses, but a unified approach to smoothing in both time and space which avoids this issue would be ideal.

Space-time analysis of individual-level emergency department data poses some other challenges as well. Investigating a specific illness among cases requires information on individuals without that illness, or controls, and with ED data, the choice of controls is not obvious. To serve as appropriate controls, they must represent the underlying population that gave rise to the cases [16]. An additional constraint of the emergency department data is that the reason for the ED visit among the controls should be unrelated to whatever may have caused the health outcome of interest among the ill. In studies with primary collection of emergency department data, participants with injuries have been used as controls for outcomes that are non-injury related [17]; however, these injured individuals may not appropriately represent the underlying population that gave rise to the cases. It may be more likely that an individual presenting with symptoms of one illness will also visit the emergency department for symptoms of an unrelated illness. Furthermore, in analyses of secondary data such as these using existing ED data of infectious diseases, injury outcomes are not collected.

## Methods

Generalized additive models are a form of non-parametric/semi-parametric regression with the ability to analyze binary or continuous outcome data while simultaneously adjusting for covariates [1]. The methods we propose use smooth terms to model space for overlapping periods of time, and covariates are controlled for parametrically to minimize data requirements. We use a loess smooth, a locally-weighted regression smoother that adapts to changes in data density, which is particularly useful when working with population-based data [1]. GAMs may exhibit biased behavior at the edges of data, and a benefit of a loess smooth is that it uses a tri-cube weight function that down-weights points far from the target point, suggesting smaller edge effects than for nearest neighbor smoothers with the same span. The amount of smoothing performed by loess is determined by minimizing the Akaike's Information Criterion (AIC). The AIC approximates the deviance-based cross validation using the average deviance of a model penalized by the number of degrees of freedom. Given an emergency department dataset of longitude, latitude, day of the ED visit, case status, and covariate age, we use longitude and latitude to model space and day to model time. Case status is the health outcome of interest and can be any illness or symptom presented for emergency care.

**Figure 1:**
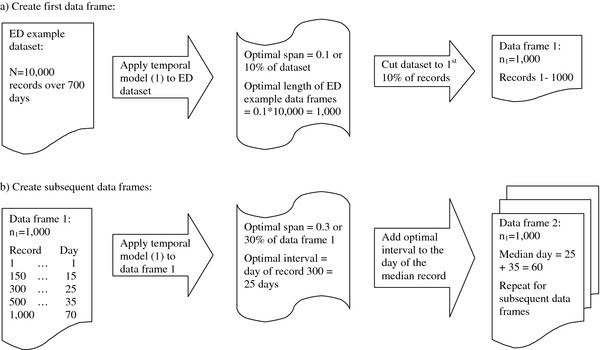
**Overlapping Data Frames.** The flow chart shows how data frames of overlapping time periods are created. The first step (**a**) is to determine the optimal length of the data frames using the optimal span of the temporal analysis for the entire ED dataset. Once the first data frame is created, the next step (**b**) is to use the optimal span of the temporal analysis for the first data frame to determine the optimal time interval between the median days of each data frame.

We first examined the ED dataset temporally using a univariate smooth (S) of time (t) measured in days

(1) $$\text{logit}\left[\text{p}\left(\text{t}\right)\right]=\text{S}\left(\text{t}\right)+\gamma 'z$$

where the left-hand side is the log of the illness odds at time (t), **z** is a vector of covariates (in our example, age), and **γ** is a vector of parameters. We used the AIC to select the statistically optimal degree of smoothing, also referred to as the optimal span size, which represents the proportion of the data used in the smooth window [1]. We then divided the ED dataset into smaller data frames of overlapping time periods based on the optimal span size from the temporal analysis [2]. By dividing the dataset into data frames of overlapping time spans, and then performing a spatial analysis within each of those time spans, we essentially smoothed over time. The optimal length of each data frame is simply the size of the smoothing window from the temporal model (1). Figure 1 illustrates how a hypothetical dataset is divided into data frames of overlapping time spans. For a span of 0.1 (or 10% of the data points), an ED dataset of 10,000 records will have an optimal length of 1,000 records in each data frame, the first data frame consisting of records 1 to 1,000 (Figure 1a). 

For the purposes of understanding illness patterns, data frames should be spaced far enough apart in time so that new cases have occurred but with sufficient overlap of data for smoothing purposes. To determine the optimal time interval between subsequent data frames, we use the optimal span for model (1) of the first data frame. The optimal interval is calculated as the number of days corresponding to the n^th^ record, where n is the length of the optimal smoothing window for the first data frame. For the hypothetical first data frame with 1,000 records, if we determine the smoothing window to be 0.3 (or 30% of the data points), then the day corresponding to record 300 (30% of 1,000 records) is the optimal interval. The optimal interval is then added to the median day of the first data frame (i.e., the day corresponding to the median record) to arrive at the median day of the second data frame. Again, for the hypothetical first data frame, if the days corresponding to records 300 and 500 (the median record) are day 25 and day 35, then the median day for the second data frame is day 35 plus an interval of 25 days, or day 60 (Figure 1b). The second data frame is created by adding records from days before and after the median day of the second data frame until the optimal length is reached. We repeat the process until the last day of a data frame is also the last day in the ED dataset.

We then examined spatial variation of each time-span data frame using a bivariate smooth (S) of longitude (x_1_) and latitude (*x*_2_)

(2) $$\text{logit}\left[\text{p}\left({\text{x}}_{1},{\text{x}}_{2}\right)\right]=\text{S}\left({\text{x}}_{1},{\text{x}}_{2}\right)+\gamma 'z$$

where the left-hand side is the log of the illness odds at location (x_1_*x*_2_), **z** is a vector of covariates (in our example, age), and **γ** is a vector of parameters [4]. We then used the AIC to select the statistically optimal degree of spatial smoothing. We created a rectangular grid of points covering the study area using the minimum and maximum longitude and latitude coordinates for the ED dataset as its dimensions. We used ArcGIS to clip grid points lying outside the outline map of the study area, in areas where people cannot live (e.g., national parks), and in areas with low population density along the edges of the dataset. Because GAMs may exhibit biased behavior at the edges of the data [1], we do not predict our spatial models in areas of low population density along the geographic edges of our study area [3]. We used model (2) to estimate the adjusted log odds at each grid point on the study area map. The same prediction grid was used for all spatial analyses, and all analyses were adjusted for age.

GAMs also provide a framework for hypothesis testing. For each time-span data frame, we first tested the global null hypothesis that case status does not depend on the smooth term using the difference of the deviances of model (2) with and without the smooth term. We estimated the distribution of the global statistic under the null hypothesis using a permutation test [4]. We ran the GAM using the optimal span of the data frame and computed the deviance statistic. A p-value cut off of 0.005 was used as a screening tool for possibly meaningful associations. Our previous work suggested the global test may have an inflated type 1 error rate when applied with a significance cut-off of 0.05; a cut-off of 0.025 provided an appropriately sized test [18]. Because a separate spatial analysis is performed on each time-span data frame, we applied an even more conservative cut off of 0.005 to account for the multiple analyses. Despite this adjustment for multiplicity, our results should be considered hypothesis generating rather than hypothesis confirming. We discuss results as “significant” if the associated p-values are less than 0.005, but acknowledge that some results still may be due to chance. If the global deviance test indicated that the map was unlikely to be flat, we next located areas of the map that exhibited clusters of unusually high or low odds of illness. We examined point-wise departures from the null hypothesis of a flat surface using the same set of permutations we used for calculating the global statistics. Once we had a distribution of log odds at every point, points that ranked in the lower and upper 2.5% of the point-wise permutation distributions were identified as areas of significantly decreased odds (“cold spots”) and increased odds (“hot spots”).

The resulting log odds from our spatial analyses were converted to odds ratios (ORs) using the whole study population as the reference, dividing the predicted odds by the odds calculated by the reduced model while omitting the smooth term. Adjusted ORs and pointwise permutation test ranks were exported from R [19] into ArcGIS [20] for mapping. R-code and simulated data are freely available [21]. The results for each time-span data frame were displayed using the same dark blue to dark red continuous color scale and the same range of odds ratios to make maps visually comparable. We denoted cold and hot spots on the maps using black contour lines created from the pointwise permutation test ranks. Maps were saved as image files and used to create a storyboard in Windows Movie Maker [22]. Each map plays for 0.5 seconds before transitioning to the next map. The resulting movie shows how illness risk varies over space and time.

To illustrate our methods with a real-world application, we used daily ED data from the Cape Cod region of Massachusetts, collected by the Institute for Health Metrics from three Cape Cod hospitals. Each record in the dataset includes the geocoded patient billing address (in longitude and latitude), the date of the emergency department visit, the patient’s age, and the syndrome group of the patient’s complaint [5]. We converted date to a continuous measure of time by setting the first record to day 1 and calculating subsequent days based on the days that passed between records. We used the optimal span sizes for the temporal analysis of the entire dataset (0.03) and the first time-span data frame (0.25) to divide the dataset into a series of overlapping time-span data frames. For this application, we examined the space-time distribution of gastrointestinal illness in Cape Cod using the proposed methods [23]. Patients who presented with respiratory illnesses were chosen as a control group. The respiratory illnesses displayed a consistent temporal pattern during the study time period with expected increased numbers in the winter season and geographic distribution that was comparable to population distributions from census data. When controls are appropriately sampled from the population giving rise to the cases, the case–control ratio (illness odds) in a subset of the area should be proportional to the incidence rate [16]. The IRB for the Boston University Medical Campus approved this research.

## Results

**Figure 2:**
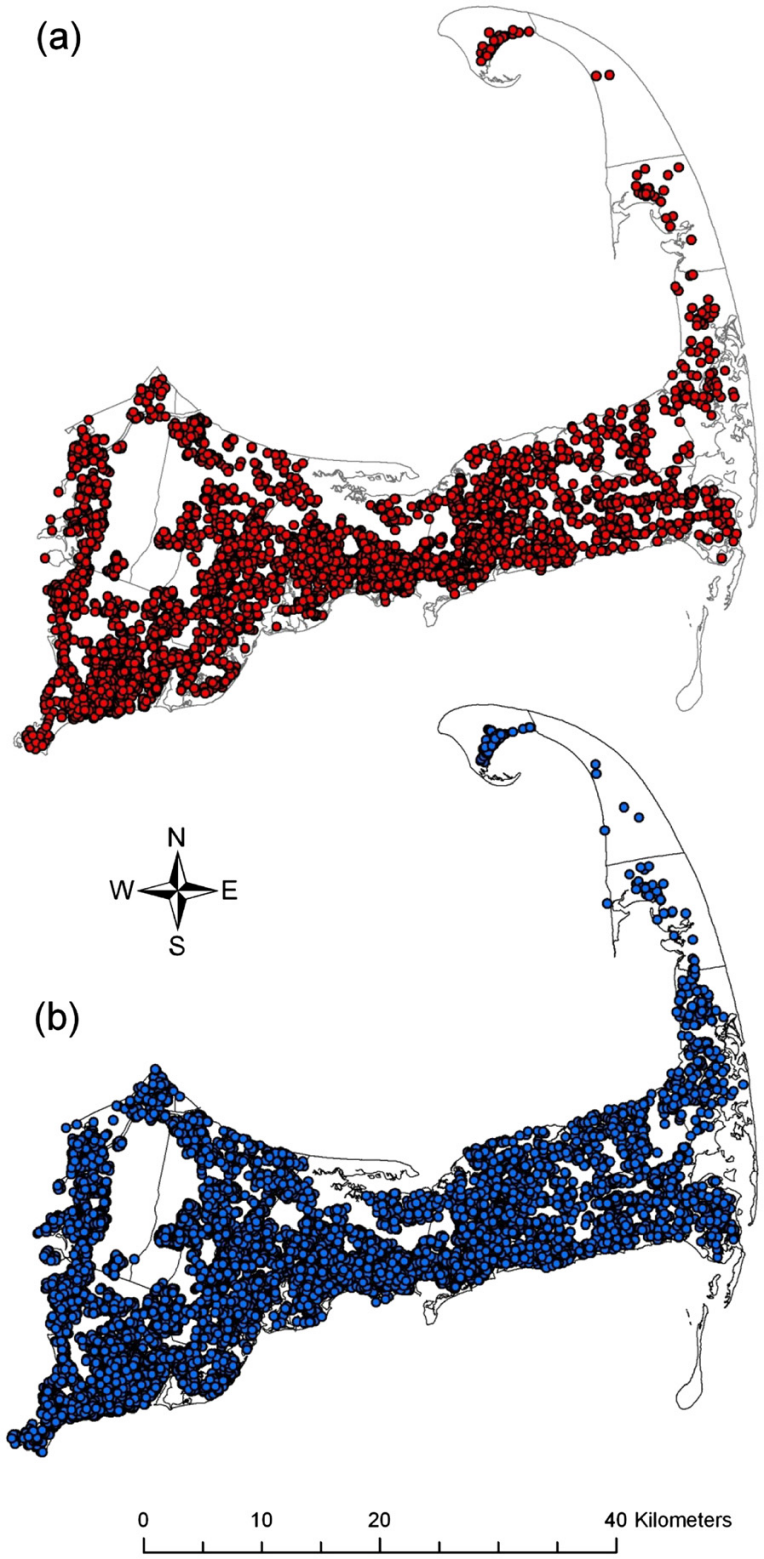
**Cape Cod Study Area.** Cape Cod is located in Massachusetts in the northeast United States. Each point represents the geocoded billing address of one participant. The distribution of (**a**) the cases with gastrointestinal illness is similar to that of (**b**) the controls with respiratory symptoms. Locations have been geographically altered to preserve confidentiality.

**Figure 3:**
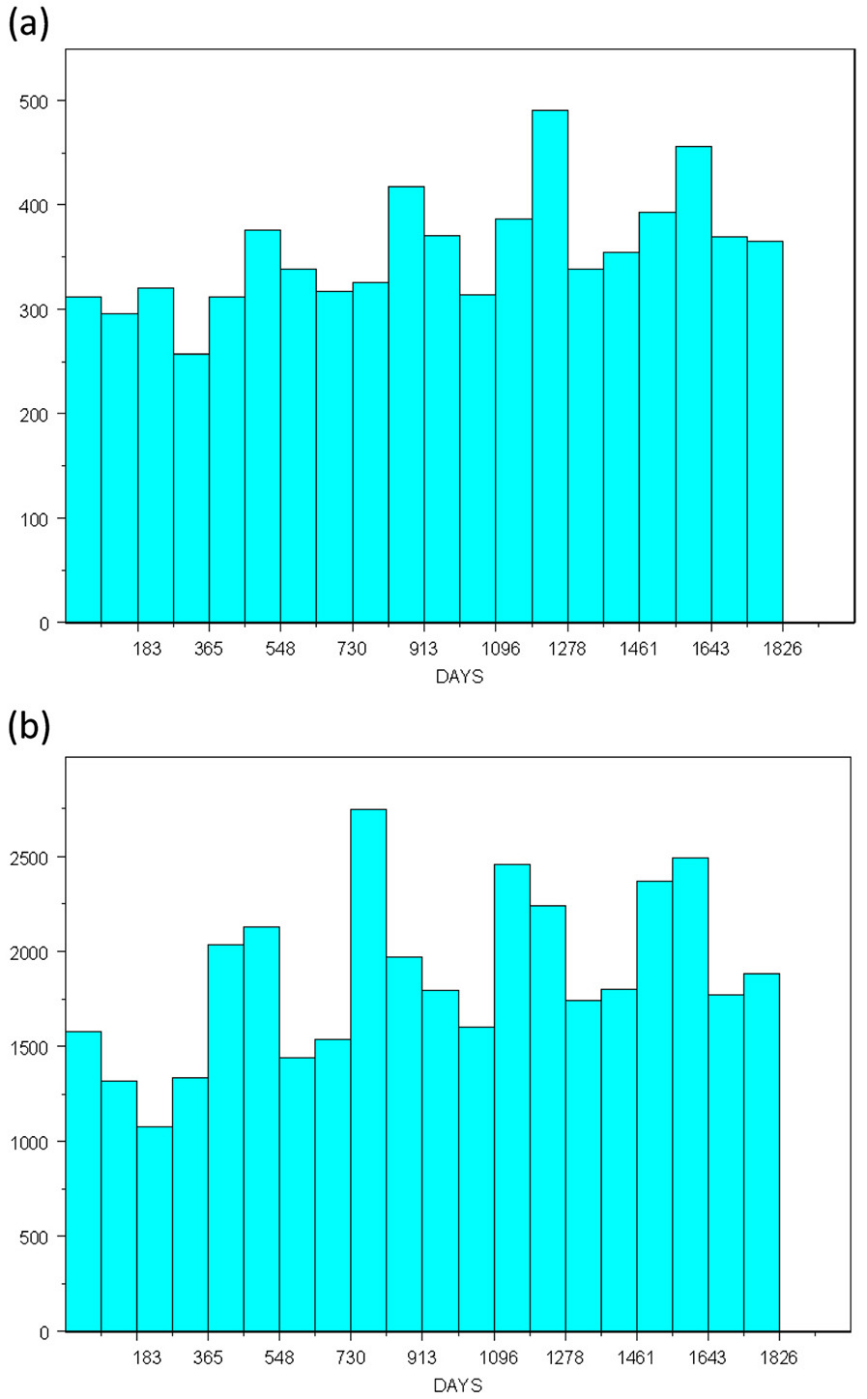
**Temporal Distribution of ED data.** The temporal distribution of (**a**) the cases with gastrointestinal illness compared to that of (**b**) the controls with respiratory symptoms. The controls show a consistent temporal pattern during the study time period with expected increased numbers in the fall and winter seasons.

We applied our methods to a subset of ED data collected over a 5 year period (1826 days) that included 7,111 cases of gastrointestinal illness and 37,310 patients with respiratory symptoms who served as controls. Patients’ ages ranged from newborn to 106 years, with a median age of 31 years. Figure 2 shows that the underlying spatial distribution of the controls is similar to that of the cases. Locations were slightly altered to preserve confidentiality. The temporal distribution of the GI cases and respiratory syndrome controls are presented in Figure 3. When we applied a univariate smooth of time measured in days, the optimal span was 3% of the data. The ED dataset contains 44,421 records, so each time-span data frame was a minimum of 1,333 records. The median date for the first data frame is day 46 and the optimal time interval of 14 days between data frames was calculated using the optimal span of 25% for a temporal smooth of the first data frame alone. Beginning with median day 46, we created data frames at 14 day intervals until median day 1810, at which point the last record of the ED dataset was included, for a total of 127 data frames of overlapping time periods.

We created a movie of continuous space-time animation for gastrointestinal illness based on location and time (see Additional file 1). The movie begins with the first data frame (median day 46, which for simplicity is labeled Week 1 in the movie) as the first frame of the movie and moves two weeks at a time until the last data frame (median day 1810, Week 257), for a total of 127 map frames. Each frame of the movie shows the risk of GI illness in the study area for that time period, the p-value for the global hypothesis test, and black contour lines depicting areas of statistically significant high or low odds ratios when applicable. Risk was not predicted for the military reservation in the west, conservation area in the north, and area of low population density to the far northeast region of the study area.

**Figure 4:**
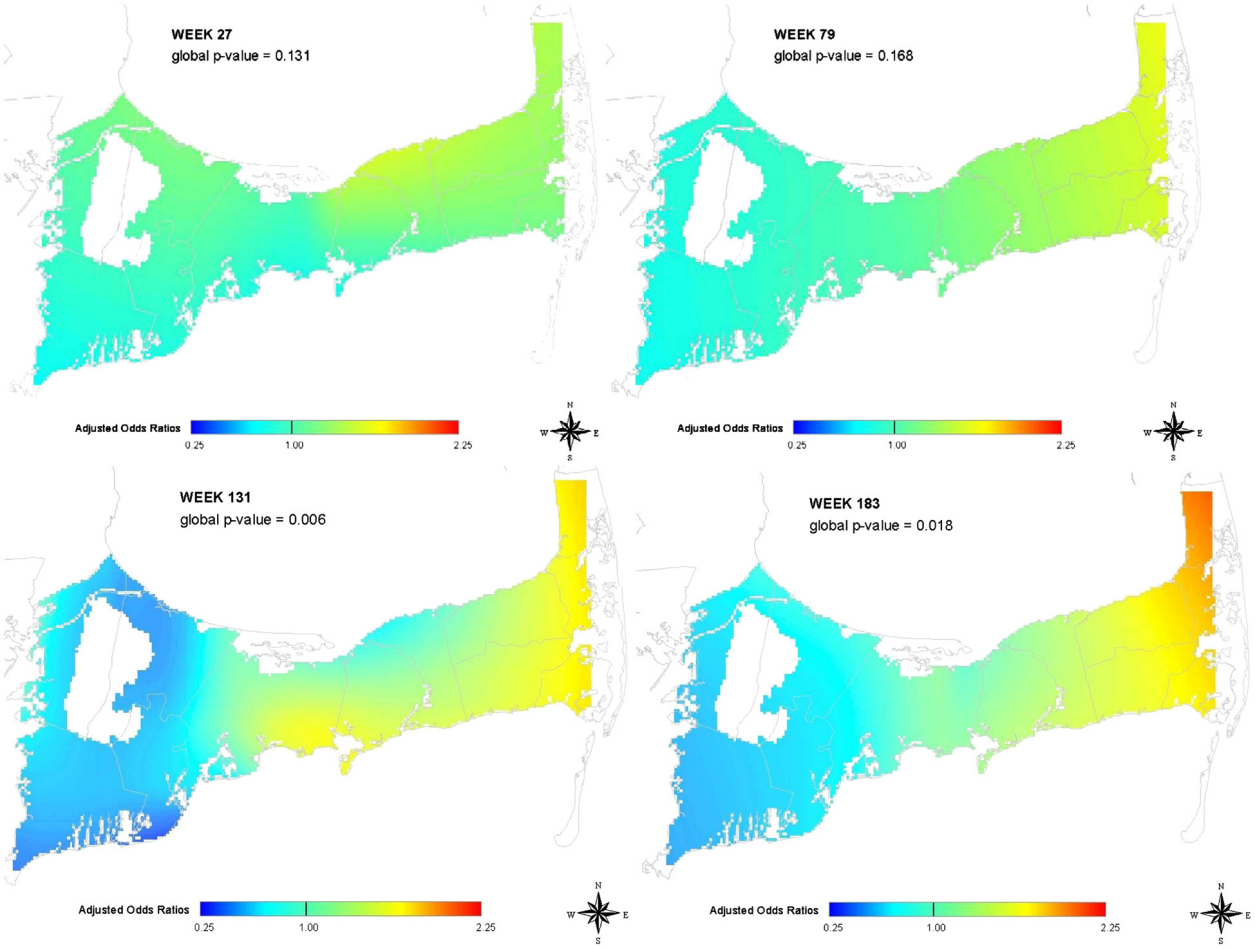
**Geographic patterns of gastrointestinal illness in Cape Cod, MA using a space-time analysis of emergency department data.** Selected map frames show similar annual patterns of GI illness based on patients’ address and date of emergency department visit. Optimal spans for these maps were at least 50% of the data.

From the movie, we observed a predominant underlying spatial pattern of higher gastrointestinal illness risk in the mid and eastern Cape Cod towns and lower risk in the western Cape Cod towns. It is important to note that the results must include both areas of increased and decreased risk as the average log odds of the entire study area is used for calculating odds ratios. In Figure 4, selected annual maps illustrate that this underlying pattern is fairly consistent. The optimal span sizes for these maps are large, usually at least 50%, suggesting a widespread trend rather than an isolated geographic occurrence.

**Figure 5:**
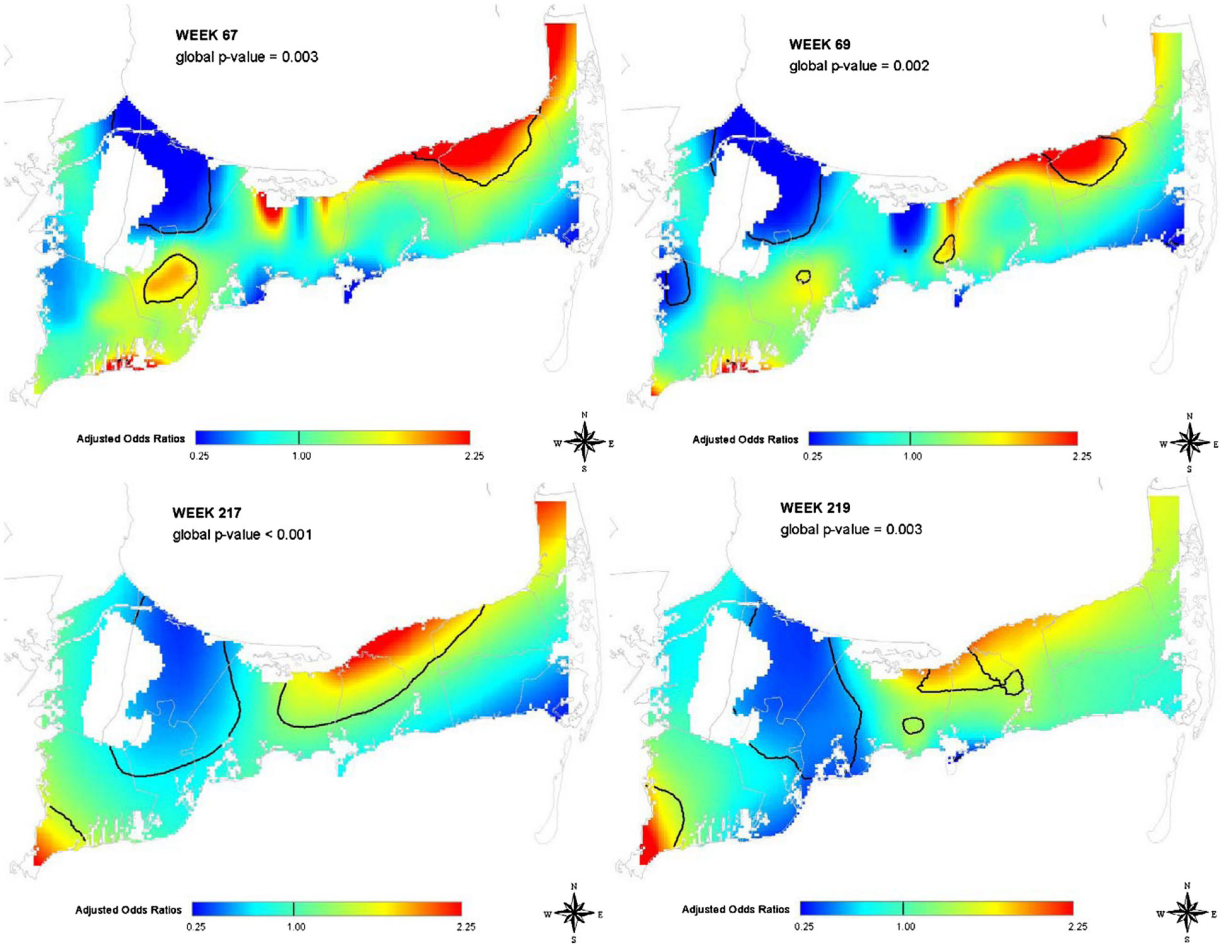
**Spatial variation of gastrointestinal illness in Cape Cod, MA using emergency department data.** Selected map frames show changing patterns of GI illness over space and time based on patients’ address and date of emergency department visit. Optimal spans for these maps were all less than 50% of the data.

Variations in the underlying pattern were also observed. Figure 5 shows weeks with significant increased risk observed in study areas other than the expected eastern region. These patterns are more geographically isolated and may indicate areas where further investigation may be warranted.

## Discussion

Space-time maps allow for pattern analysis of illnesses among cases and controls using individual-level data [24-33]. The GAM method visualizes risk while adjusting for known confounders and testing for the statistical significance of location and time. Our analyses illustrate its application as a method for secondary retrospective analysis of existing ED data. No one method is ideal for every disease cluster investigation and each contributes different and important features to space-time analyses [1-14]. While many methods focus on identifying disease outbreaks, we were primarily interested in visualizing the broader underlying pattern of disease.

Although GAMs have many advantages, a number of issues remain. The GAM analysis uses separate optimal smoothing spans for time and space based on minimizing the AIC for each dataset. While this ensures that the appropriate span is used in each map, it does sometimes result in sudden and spatially large-scale changes. This is less problematic in our application for exploratory analyses, but ideally, we would like to use smoothing spans determined to be optimal in a combined time-space framework so that the method is useful for other spatio-temporal applications as well. Further work is needed to resolve this methodological issue, and we are currently exploring additional GAM methods for simultaneously smoothing time and space [15]. Until such statistical methods have been evaluated and modified for use with public health data, the methods presented here are appropriate for exploratory space-time analyses. The ease of implementation using existing standard software makes it accessible to other researchers and practitioners. GAMs may also exhibit edge effects, which are biased behavior at the edges of the data [1]. As much of our spatial data is found along the edges (i.e., population is denser by the coastline), this issue remains a concern despite our work with synthetic data showing little, if any, edge effect [4]. In the current space-time analysis, we restricted the prediction of risk to areas with high population density in order to limit some of the edge effect bias that may occur.

We computed global and pointwise p-values, but many epidemiologists prefer confidence intervals when evaluating the precision of point estimates [16]. It should be possible to compute variance bands (also known as confidence bands) for our maps using bootstrap methods and work on this, including how to display variance bands in our movies, is ongoing [1]. Further, the global and pointwise hypothesis tests result in having to make multiple comparisons, which increases the likelihood of finding location significant by chance alone. To adjust for multiplicity, we use a conservative global p-value and we only conducted pointwise tests if the global deviance test indicated that the map was unlikely to be flat. The location of significant hot and cold spots should be considered exploratory. As our objective is to measure the geographic patterns of disease rather than identify specific clusters, we focused our results on comparison of magnitude and location of risk regardless of statistical significance. Rather than using statistical significance to identify clusters, another approach is to use a predefined risk level based on the outcome of interest and use that as a way to determine public health significance [10]. This is something we will pursue in future work.

Secondary analyses using existing surveillance data also has its own set of challenges. Further work is needed to determine the optimal control group in a surveillance dataset. There is a possibility that variations in patterns of the control group would result in a shifting reference, for example temporal variations in our controls that do not reflect changes in the population giving rise to the cases. We are currently exploring the use of cases from a prior time period as the reference. While areas of increased or decreased risk may theoretically be caused by non-uniform control selection, selection of controls within the study area did not depend on geography. Also, surveillance data do not include extensive covariates so the observed patterns may be due to spatial confounders like socioeconomic status. Despite these limitations, GAMs remain a useful framework for examining spatial and temporal patterns from ED datasets.

## Conclusions

The GAM methods we presented predict risk of illness with a bivariate smooth for longitude and latitude using data frames of overlapping time periods to smooth over time while simultaneously adjusting for confounders. We illustrated this approach using existing surveillance data for the Cape Cod region of Massachusetts. By spatially analyzing data frames created from records of overlapping time periods, we were able to create a movie that showed geographic patterns of illness that were generally similar over time but showed significant variation at certain time periods. The public health of the community during these time periods may warrant further investigation.

## Competing interests

The authors declare they have no competing interests.

## Authors’ contributions

VV conducted the spatial-temporal analyses and drafted the manuscript. TW collaborated on all analytical and editorial decisions. JW provided statistical support and consulted on analytical and editorial issues. All authors read and approved the final manuscript.

## Pre-publication history

The pre-publication history for this paper can be accessed here:

http://www.biomedcentral.com/1471-2458/12/687/prepub

## Supplementary Material

Additional file 1 Space-time analysis of ED data. Click here for file
